# Abscisic-acid-responsive *StlncRNA13558* induces StPRL expression to increase potato resistance to *Phytophthora infestans* infection

**DOI:** 10.3389/fpls.2024.1338062

**Published:** 2024-03-05

**Authors:** Kaijie Shang, Ruolin Wang, Weilin Cao, Xipan Wang, Yubo Wang, Zhenting Shi, Hongmei Liu, Shumei Zhou, Xiaoping Zhu, Changxiang Zhu

**Affiliations:** ^1^ College of Life Sciences, Shandong Agricultural University, Tai’an, Shandong, China; ^2^ College of Plant Protection, Shandong Agricultural University, Tai’an, Shandong, China; ^3^ The Engineering Research Institute of Agriculture and Forestry, Ludong University, Yantai, Shandong, China

**Keywords:** disease-related protein, long non-coding RNA, phytohormone, *Phytophthora infestans*, potato (*Solanum tuberosum* L)

## Abstract

Late blight, caused by *Phytophthora infestans*, is one of the most serious diseases affecting potatoes (*Solanum tuberosum* L.). Long non-coding RNAs (lncRNAs) are transcripts with a length of more than 200 nucleotides that have no protein-coding potential. Few studies have been conducted on lncRNAs related to plant immune regulation in plants, and the molecular mechanisms involved in this regulation require further investigation. We identified and screened an lncRNA that specifically responds to *P. infestans* infection, namely, *StlncRNA13558*. *P. infestans* infection activates the abscisic acid (ABA) pathway, and ABA induces *StlncRNA13558* to enhance potato resistance to *P. infestans*. *StlncRNA13558* positively regulates the expression of its co-expressed PR-related gene *StPRL*. StPRL promotes the accumulation of reactive oxygen species and transmits a resistance response by affecting the salicylic acid hormone pathway, thereby enhancing potato resistance to *P. infestans*. In summary, we identified the potato late blight resistance lncRNA *StlncRNA13558* and revealed its upstream and downstream regulatory relationship of *StlncRNA13558*. These results improve our understanding of plant-pathogen interactions’ immune mechanism and elucidate the response mechanism of lncRNA-target genes regulating potato resistance to *P. infestans* infection.

## 1 Introduction

During their long-term evolution, plants have gradually developed a series of complex and effective protection mechanisms to resist infection by pathogenic microorganisms. With the rapid development of potato disease resistance breeding technology since the 20th century, researchers have deeply analyzed the mechanism of potato disease resistance from different perspectives, such as potato structural resistance, reactive oxygen species changes, changes in defense enzymes, and various signal molecules involved in disease resistance and their mediated signal transduction pathways, and cloned and identified numerous disease resistance genes or factors (Valkonen, 2015; Chang et al., 2020; Ivanov et al., 2021). The discovery of potato disease resistance genes has accelerated the development of omics strategies in recent years (Kwenda et al., 2016; Duan et al., 2020). In addition to disease resistance genes, certain long non-coding RNAs (lncRNAs) may play an important role in the biological process of plant resistance to pathogen infection (Burkhardt and Day, 2016; Zaynab et al., 2018).

lncRNAs are RNA transcripts with a length of more than 200 nucleotides and no obvious protein-coding potential (Sun et al., 2018). Only a small part of the genome is transcribed into mRNA encoding proteins, most of which produce several lncRNAs (Derrien et al., 2012). During plant growth and abiotic stress response, plant lncRNAs play key roles in auxin transport, flowering time, phosphate signaling, root organogenesis, and fruit ripening (Ariel et al., 2014; Li et al., 2015; Wang et al., 2017; Kim et al., 2019; Yu et al., 2019). The study of lncRNAs related to plant immune regulation is increasingly becoming a hot spot. The transcription factor WRKY1 can induce the accumulation of H_2_O_2_ and participate in the defense mechanism of tomatoes against *Phytophthora infestans* by activating the *lncRNA33732* expression (Cui et al., 2019). Rice lncRNA ALEX1 can improve the rice resistance to bacterial wilt by activating the jasmonic acid pathway (Yu et al., 2020). The *LncRNA39026* in tomatoes positively regulates their defense response to *P. infestans* by regulating the SlAGO1 expression as an endogenous target of miR168a (Hou et al., 2020). The knockout of *SllncRNA39896* promotes the accumulation of miR166b and increases the cleavage of *SlHDZ34* and *SlHDZ45* transcripts, which enhance the resistance of tomatoes to *P. infestans* (Hong et al., 2022). Pathogen infection reduces the accumulation of immune negative regulatory factor lncRNA SABC1, which in turn relieves the inhibition of the salicylic acid (SA) synthase expression and promotes SA synthesis to enhance plant immunity (Liu et al., 2022). The functions and molecular mechanisms of lncRNAs in plant immune responses require further study.

Pathogenesis-related (PR) proteins are water-soluble proteins produced by plants following pathogen infection or abiotic stress (Van Loon and Van Strien, 1999). PR-1 proteins are often used as markers for pathogen-induced systemic acquired resistance (SAR) to enhance the defense status (Van Loon et al., 2006). Many PR proteins are accumulated in potato leaves infected by *P. infestans*. Among them, PR-1b, which is the protein with the most abundant induced expression, was purified and identified as a new member of the PR-1 protein family (Hoegen et al., 2002). PR-10, a protein with antifungal properties, was significantly upregulated during infection with *Fusarium thapsinum* and *Curvularia lunata* (Katilé et al., 2010). The PnPR-like gene in the traditional Chinese herbal medicine *Panax notoginseng* responds to the infection of the root rot pathogen *Fusarium solani*; its expression is induced by various signal molecules, such as jasmonic (JA), ethylene (ET), and SA, and it has an antifungal activity (Li et al., 2020). The secreted PR1 protein has an anti-ovarian activity and can be transferred from the host to the pathogen. The translocated PR1 protein targets the protein kinase AMPK in *P. infestans*, thereby reducing the phosphorylation of downstream proteins driven by AMPK, in turn inhibiting the pathogen’s vegetative growth and pathogenicity (Luo et al., 2023).

Abscisic acid (ABA) is important in plant growth, development, and environmental stress (Waadt et al., 2022). Overexpression of the wheat TaPYL1-1B gene increased ABA sensitivity, photosynthetic capacity, and water use efficiency, thereby enhancing drought tolerance in wheat (Mao et al., 2022). ABA is a defensive hormone and an important component of the plant immune system (Mauch-Mani and Mauch, 2005; Sánchez-Vallet et al., 2012). Exogenous ABA can enhance tomato resistance to *Alternaria solani* by activating defense gene expression and enhancing defense-related enzyme activities (Song et al., 2011). miRNAs were identified after ABA treatment of tomatoes; most of the miRNAs were downregulated, and the corresponding disease resistance genes were upregulated (Cheng et al., 2016). The knockout of CmMLO17 in *Chrysanthemum morifolium* enhances its resistance to *A. alternata*. Resistance-related genes were differentially expressed in the knockout lines, and ABA and Ca^2+^ signaling pathway genes were upregulated (Xin et al., 2021). Our previous studies showed that ABA can regulate stomatal opening and closing during pathogen infection and enhance potato resistance to *P. infestans* (Wang et al., 2021).

Potatoes are one of the most important food crops worldwide; however, they can be infected with various pathogens during growth and development (Fones et al., 2020). Potato late blight caused by *P. infestans* is one of the most serious diseases in potatoes, which seriously affects the yield and quality of potatoes (Haverkort et al., 2009; Ivanov et al., 2021). The selection of disease-resistant varieties is the most economical, effective and environmentally friendly method to control late blight, but no potato varieties with complete resistance to late blight have been found so far. To fully explore the genes or factors of potato resistance to *P. infestans* and deeply analyze the mechanism of disease resistance can provide important gene resources and theoretical basis for crop disease resistance molecular breeding, which is an important basis for developing green and efficient crop disease prevention and control technologies.

## 2 Materials and methods

### 2.1 Plant materials and growth condition

Aseptic tissue culture seedlings of potato (*S. tuberosum* L.) and *Nicotiana benthamiana* seeds were preserved in Shandong Agricultural University in Tai’an, China. Two-week-old sterile potato tissue culture seedlings were transplanted into the nutrient-rich soil. The seeds of *N. benthamiana* were spread on MS medium for germination and transplanted into nutrient-rich soil. The stable transformation of potatoes was carried out using the leaf disc transformation method as previously described (Peng et al., 2022). The plants were transplanted into nutrient-rich soil after transformation. The plants were grown in a temperature-controlled greenhouse. The growth conditions were 24°C and photoperiodic lighting (16 h of light:8 h of dark).

### 2.2 Sequence analysis

The online software PLANT CARE (http://bioinformatics.psb.ugent.be/webtools/plantcare/html/) was used to predict the cis-acting elements of the promoter, and the parameters were set according to the default settings of the webpage. Primer specificity was checked against the NCBI for Biotechnology Information (https://www.ncbi.nlm.nih.gov/) database using the Primer-BLAST program. The transcriptome sequence read archive of the NCBI (https://www.ncbi.nlm.nih.gov/) collection was used to obtain potato RNA data sets (PRJNA203403), which were used to identify potato transcripts in response to *P. infestans* infection.

### 2.3 Plasmid construction and *Agrobacterium* infection

According to the plasmid construction method used in our laboratory (Shang et al., 2023), the *StlncRNA13558* gene was ligated to the restriction site region between the 35S promoter and Nos terminator of the pCa plasmid to obtain the pCa-*StlncRNA13558* overexpression plasmid. The *StPRL* gene was ligated to the restriction site region between the 35S promoter and the Nos terminator of the pCa-GFP plasmid to obtain the pCa-StPRL-GFP overexpression plasmid. The *StlncRNA13558* and *StPRL* genes were ligated to the restriction site region between the 35S promoter and the Nos terminator of the TRV2 plasmid to obtain the TRV2-*StlncRNA13558* and TRV2-StPRL downregulated plasmids. Empty pCa, pCa-GFP, and TRV2 plasmids used in this study were preserved in our laboratory.

The recombinant plasmid used for *Agrobacterium* infection was transformed into *Agrobacterium* GV3101. The *Agrobacterium* infection solution (10 mM of MgCl_2_, 10 mM of MES [pH 5.6], and 100 μM of acetosyringone) was prepared, and the *Agrobacterium* was diluted to the working concentration of OD600 = 0.5. A sterile syringe was used to infect the abaxial sides of the third expanded leaf of four-week-old *N. benthamiana* to infect *N. benthamiana* instantaneously. This technique was also used to infect the abaxial sides of the fully expanded leaves of four-week-old potatoes.

### 2.4 Inoculation of *P. infestans* and measurement of lesion diameter

*P. infestans* strains preserved in our laboratory were inoculated on rye medium and cultured at 18°C in the dark for approximately two weeks. When the mycelia of *P. infestans* covered the plate, sterile distilled water was added, and the mycelia were removed using a coating rod. Hyphae were transferred to a centrifuge tube using a pipette and incubated in a refrigerator at 4°C for 4 h. Spores were counted using a blood cell counter. The number of spores of the *P. infestans* was adjusted to 15000–20000 Sporangia/mL with sterile distilled water, and a spore suspension of *P. infestans* was obtained.

When the entire plant was inoculated, the spore suspension of *P. infestans* was uniformly sprayed onto one-month-old potato plants, while the control group received a water treatment. Each treatment was performed in triplicate and cultured in a light room. Detached leaves were inoculated on an inoculation plate by adding 30 μL of the *P. infestans* spore suspension to the abaxial sides of the leaves with a pipette. The inoculation plate was placed in an 18°C constant-temperature incubator with photoperiodic lighting (16 h of light:8 h of dark). Diseased leaves were observed 5 d after inoculation, and the diameters of the lesions were measured using a scale. Ten leaves were used for each experiment. All experiments were performed in triplicate.

### 2.5 RNA extraction and qRT-PCR assay

A total of 0.5 g of plant materials was ground in liquid nitrogen, mixed with 1 mL of TRIzol (Invitrogen, Shanghai, China), and then extracted with 200 μL of chloroform. After centrifugation at 12000 rpm for 10 min, the supernatant was collected and mixed with an equal volume of isopropanol. The precipitated RNA precipitation was obtained after centrifugation at 12,000 rpm for 10 min. The RNA was extracted after washing with 75% ethanol. RNA was reverse-transcribed into cDNA (Vazyme, Nanjing, China, R333), and qRT-PCR was performed according to the SYBR Mix instructions (Vazyme, Nanjing, China, Q411). qRT-PCR was performed using LightCycler96 (Roche, Rotkreuz, Switzerland). The potato *EF1α* gene and the *N. benthamiana actin* gene were used as the internal reference genes. The internal reference gene and detection primers used in qRT-PCR are shown in **Supplementary Table 1**. Data were collected from three biological replicates, and the bar value represents the standard deviation. Statistical analysis of the two sets of data was based on the Student’s t-test, ^ns^ *p* > 0.05, *p < 0.05, **p < 0.01, ***p < 0.001, and ****p < 0.0001.

### 2.6 Subcellular localization of StPRL

The pCa-StPRL-GFP overexpression plasmid was transformed into *Agrobacterium* to infect *N. benthamiana*. The infected area was removed 3 d after infection to prepare observation slides, stained using 4′,6-diamidino-2-phenylindole (DAPI) staining solution, and observed using a laser confocal microscope (LSM 880; Zeiss, Oberkochen, Germany). Green fluorescent protein (GFP) was excited at 488 nm and captured at 510–550 nm. DAPI was excited at 350 nm and captured at 460 nm using a 20× objective lens. Ten independent plants were analyzed. All experiments were performed in triplicate.

### 2.7 Western-blot analysis of StPRL

The plant materials were ground in liquid nitrogen; 0.1 g was weighed and placed in a centrifuge tube, and the total protein was extracted by adding 100 μL protein extraction liquid (CWBIO, Beijing, China). The samples were loaded onto a 12.5% polyacrylamide gel and subjected to electrophoresis for 1.5 h at a constant voltage of 150 V. The gel was transferred to a membrane at a constant current of 200 mA for 1 h, and the protein bands were transferred to a polyvinylidene fluoride (PVDF) membrane. The proteins were detected using chemiluminescence detection with GFP- and secondary antibodies and photographed using an *in vivo* imager.

### 2.8 3, 3’-Diaminobenzidine staining of *N. benthamiana* leaves

DAB powder (0.1 g) was added to 100 mL of sterile water, and the pH was adjusted to approximately 3.8 with NaOH. The treated leaves were cut into discs with a diameter of 1 cm, immersed in the DAB solution, vacuum-filtered for 30 min, and stained at 20°C in the dark for 16 h. The staining solution was discarded, and the fixation solution (anhydrous ethanol:lactic acid:glycerol = 3:1:1) was added to the submerged leaves and make it boiled in water for 20 min until the leaf green completely faded. The specimen was cooled to room temperature and rinsed with 75% alcohol, and the staining developed. All experiments were performed in triplicates.

### 2.9 Statistical analysis

Data were collected from three biological experiments. All statistical analyses were implemented in GraphPad Prism v.7.0, using Student’s two-tailed t-test. Differences were considered significant at p < 0.05.

## 3 Results

### 3.1 *P. infestans* infection activates the ABA pathway and induces the *StlncRNA13558* expression in potatoes

**Figure 1:**
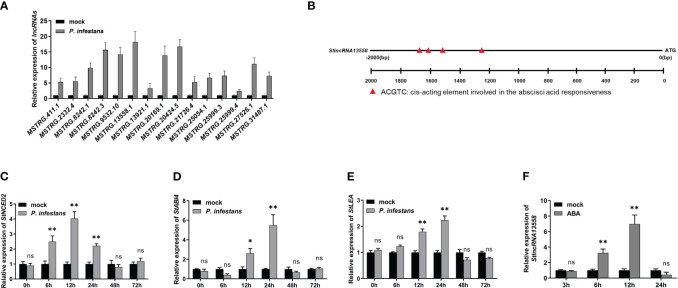
** **P*. infestans* infection activates the ABA pathway and induces the *StlncRNA13558* expression in potatoes. **(A)** The differentially expressed lncRNAs during *P. infestans* infection were detected by qRT-PCR. **(B)** Promoter cis-acting element analysis of *StlncRNA13558*. **(C–E)** The relative expression levels of ABA synthesis and transduction related genes *StNCED2*, *StABI4* and *StLEA* were detected by qRT-PCR. **(F)** The relative expression level of *StlncRNA13558* was detected by qRT-PCR. Data were collected in three biological experiments, and the Bar value represents the standard deviation. The statistical analysis was based on Student’s t test, ^ns^ *p* > 0.05, *p < 0.05, and **p < 0.01.

On the basis of previously reported transcriptome data (Cao et al., 2021), we further studied the response mechanism of potato lncRNAs in the process of *P. infestans* infection. We selected 15 lncRNAs that were differentially expressed at 6 hours after *P. infestans* infection for qRT-PCR, and compared them with the expression of lncRNAs in the simulated infection control group potatoes (**Figure 1A**; **Supplementary Table 2**). *MSTRG.13558.1*, which was highly up-regulated in the control group potatoes and potatoes infected by *P. infestans*, was selected and named *StlncRNA13558* for further study. We analyzed the promoter cis-acting elements of *StlncRNA13558*, selected the 2000 bp upstream of the gene as the promoter sequence, and used the online software PLANT CARE to predict the promoter cis-acting elements. The prediction results show that the promoter of *StlncRNA13558* contained ABA binding elements (**Figure 1B**).

ABA is an important regulator of the plant immune system (Song et al., 2011; Xie et al., 2018). We inoculated one-month-old wild-type potatoes with *P. infestans* and used water treatment as the control. The expression levels of ABA synthesis and transduction related genes *9-cis-epoxycarotenoid dioxygenase 2* (*StNCED2*), *ABA insensitive 4* (*StABI4*) and *late embryogenesis abundant protein* (*StLEA*) were detected at different time points. The expression levels of *StNCED2*, *StABI4* and *StLEA* increased first and then decreased. The expression level of *StNCED2* reached the highest at 12 h, and the expression levels of *StABI4* and *StLEA* reached the highest at 24 h (**Figures 1C–E**). We sprayed 100 μM of ABA on one-month-old wild-type potato leaves and used water treatment as the control. *StlncRNA13558* expression was detected using qRT-PCR on samples collected 3, 6, 12, and 24 h after ABA treatment. The results show that compared with the control, the *StlncRNA13558* expression increased first and then decreased after spraying ABA, and reached the highest after 12 h of ABA treatment (**Figure 1F**). These results indicated that *P. infestans* infection can promote the expression of ABA synthesis and transduction-related genes, and that ABA pathway activation induces the expression of *StlncRNA13558*.

### 3.2 *StlncRNA13558* enhances the plant resistance to *P. infestans*

**Figure 2:**
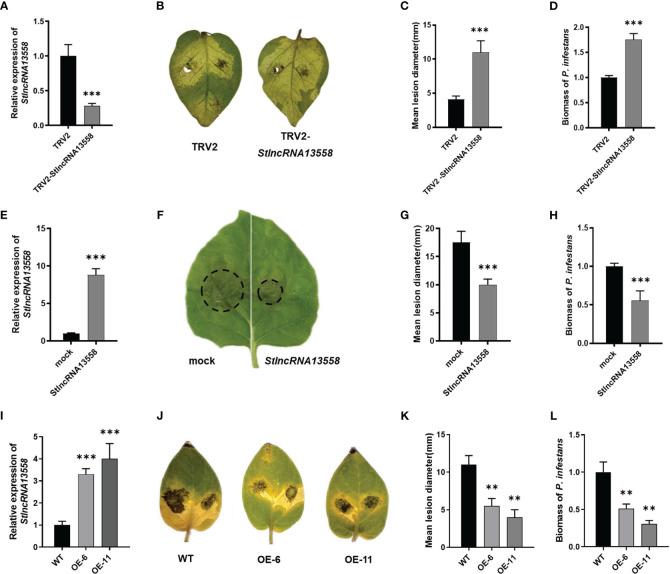
*StlncRNA13558* enhances the plant resistance to *P. infestans.* **(A)** The relative expression level of *StlncRNA13558* in the potatoes in which the *StlncRNA13558* expression was downregulated by VIGS was detected by qRT-PCR. **(B)** Representative images of *P. infestans* lesions on potato leaves in which *StlncRNA13558* was downregulated by VIGS, compared to the control. **(C)** The diameter of the lesions on the potato leaves with VIGS downregulated *StlncRNA13558*. **(D)** The relative expression level of the *P. infestans*-specific *PiO8* element was detected by qRT-PCR. **(E)** The relative expression level of *StlncRNA13558* in the transient overexpression of *N. benthamiana* was detected by qRT-PCR. **(F)** Representative images of *P. infestans* lesions on *N. benthamiana* leaves in which transiently overexpressing *StlncRNA13558*, compared to the control. **(G)** The diameter of the lesions on *N. benthamiana* leaves with the transient overexpression of *StlncRNA13558*. **(H)** The relative expression level of the *P. infestans*-specific *PiO8* element was detected by qRT-PCR. **(I)** The relative expression level of the stable overexpression of *StlncRNA13558* in the potatoes was detected by qRT-PCR. **(J)** Representative images of *P. infestans* lesions on potato leaves in which stable overexpression of *StlncRNA13558*, compared to the control. **(K)** The diameter of the lesions on the potato leaves with the stable overexpression of *StlncRNA13558*. **(L)** The relative expression level of the *P. infestans*-specific *PiO8* element was detected by qRT-PCR. The range of disease spots on the leaves of native *N. benthamiana* is encircled. Data were collected in three biological experiments, and the Bar value represents the standard deviation. The statistical analysis was based on Student’s t test, **p < 0.01, and ***p < 0.001.

We downregulated *StlncRNA13558* in potatoes using virus-induced gene silencing (VIGS) to study the role of *StlncRNA13558* in *P. infestans* infection. The qRT-PCR results show that the *StlncRNA13558* expression was significantly lower than that of the control (**Figure 2A**). Potato leaves were inoculated with a *P. infestans* by *in vitro* inoculation. The incidence of leaf infection and lesion diameters were observed. The results showed that the diameters of the lesions on potato leaves with downregulated *StlncRNA13558* were significantly larger than those in the control group (**Figures 2B, C**). We mixed the diseased leaves and sampled them to further clarify the abundance of *P. infestans* in infected leaves. The expression level of the *P. infestans*-specific *PiO8* element was detected using qRT-PCR. The mycelial growth on potato leaves with downregulated StlncRNA13558 was significantly higher than that in the control group (**Figure 2D**).

We constructed the overexpression plasmid pCa-*StlncRNA13558* and separately transformed the pCa-*StlncRNA13558* plasmid and the empty pCa plasmid control into *Agrobacterium*. Then, the *Agrobacterium* solution was injected into the epidermal cells of *Nicotiana benthamiana* leaves. RNA was extracted 48 h after infection, and qRT-PCR analysis was conducted. The results showed that the expression level of *StlncRNA13558* significantly increased (**Figure 2E**). We then performed *P. infestans* infection experiments and found that the lesion diameter and mycelial growth level of *P. infestans* in leaves overexpressing *StlncRNA13558* were significantly lower than those in the controls (**Figures 2F–H**).

We further transformed *StlncRNA13558* stable overexpression potato lines (**Figure 2I**). The two-week-old transgenic lines OE-6 and OE-11 and wild-type potato seedlings in the tissue culture bottle were transplanted into the greenhouse. After culturing them for a month, the potato leaves were taken *in vitro* and inoculated with *P. infestans*. After 5 days, the disease phenotype was observed, and the mycelial growth of *P. infestans* was detected. The lesion diameter and mycelial growth of transgenic lines OE-6 and OE-11 were significantly lower than those of the wild-type potatoes (**Figures 2J–L**). The above results indicate that *StlncRNA13558* can enhance the resistance of potatoes to *P. infestans*.

### 3.3 *StlncRNA13558* induces StPRL expression

**Figure 3:**
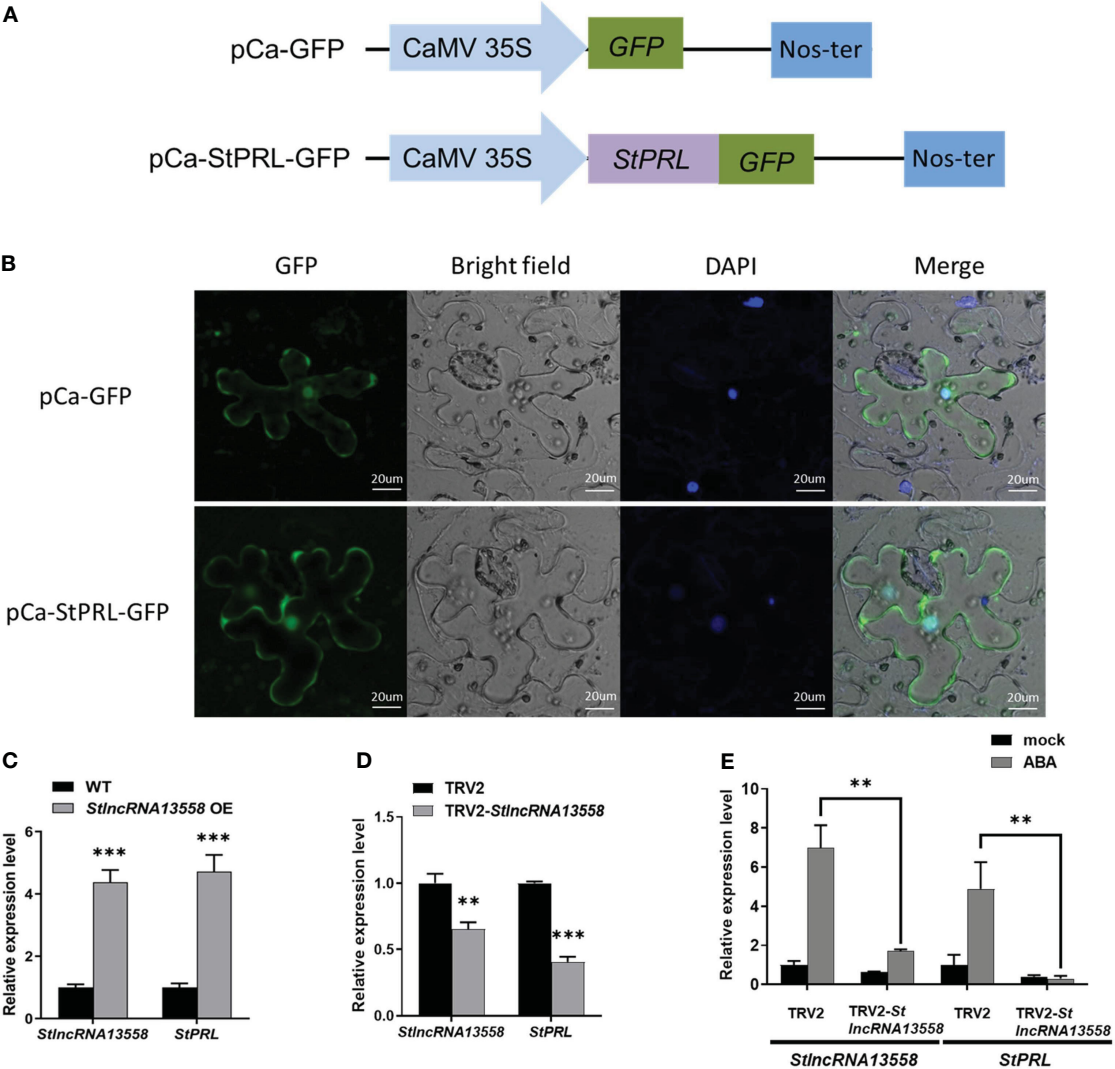
*StlncRNA13558* induces StPRL expression. **(A)** Schematic illustration of the pCa-StPRL-GFP recombinant plasmid. **(B)** The subcellular localization of StPRL was observed. From left to right, GFP, bright-field of vision, DAPI, combined field of vision, scale: 20 μm. The empty pCa-GFP plasmid was used as the control. **(C)** The relative expression level of *StPRL* in the potatoes with a stable overexpression of *StlncRNA13558* was detected by qRT-PCR. **(D)** The relative expression level of *StPRL* in the potatoes with *StlncRNA13558* down-regulated by VIGS was detected by qRT-PCR. **(E)** The effect of spraying 100 μM of ABA on the relative expression levels of *StlncRNA13558* and *StPRL* in potatoes downregulated by VIGS was detected by qRT-PCR. Data were collected in three biological experiments, and the Bar value represents the standard deviation. The statistical analysis was based on Student’s t test, **p < 0.01, and ***p < 0.001.

According to our existing research data (Cao et al., 2021), we selected a *StlncRNA13558* co-expressed gene *PGSC0003DMT400063921* related to plant disease resistance in the potato genome, named *StPRL*, for further study. StPRL is a potato PR-related family protein with unknown function. We constructed the fusion expression plasmid pCa-StPRL-GFP (**Figure 3A**). The pCa-StPRL-GFP plasmid and the pCa-GFP empty plasmid control were transformed into *Agrobacterium* and transiently infected *N. benthamiana*. After 48 h, the leaf epidermal sections were made and the subcellular localization was observed using a laser confocal microscope (LSM 880; Zeiss, Oberkochen, Germany). Green fluorescence signals were observed in the cytoplasm and nucleus of StPRL (**Figure 3B**), indicating that StPRL functions in the cytoplasm and the nucleus. qRT-PCR revealed that the *StPRL* expression in the *StlncRNA13558* stable overexpression potato lines was upregulated 4.7 times that in the wild-type potatoes (**Figure 3C**).

We used VIGS to downregulate the *StlncRNA13558* in the potatoes and then detected the *StPRL* gene expression in potatoes by qRT-PCR. Compared with the potatoes infected with TRV2 empty vector, the expression level of StPRL in the *StlncRNA13558* downregulated lines was significantly reduced (**Figure 3D**). The above experiments show that *StlncRNA13558* can positively regulate the expression of *StPRL*. Subsequently, we performed ABA treatment on potatoes with downregulated *StlncRNA13558* using VIGS, and the expression of *StPRL* in the ABA-treated downregulated lines was detected using qRT-PCR. The results show that the increase of the StPRL level caused by ABA in the potatoes with downregulated *StlncRNA13558* was significantly lower than that in the control group (**Figure 3E**). The above results indicate that StPRL may respond to ABA treatment through *StlncRNA13558*, and ABA may protect potatoes against *P. infestans* infection by regulating the *StlncRNA13558*-StPRL cascade.

### 3.4 StPRL enhances the plant resistance to *P. infestans*

**Figure 4:**
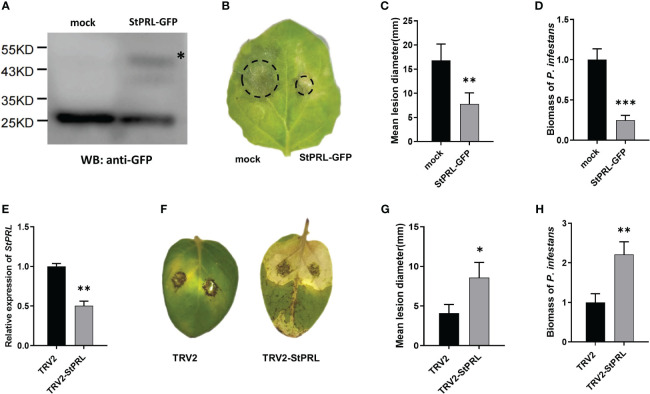
StPRL enhances the plant resistance to *P. infestans.* **(A)** The expression of StPRL in the transient overexpression of *N. benthamiana* was detected using Western blot. **(B)** Representative images of *P. infestans* lesions on *N. benthamiana* leaves in which transiently overexpressing StPRL, compared to the control. **(C)** Lesion diameter of transient overexpression of StPRL in *N. benthamiana* leaves. **(D)** The relative expression level of the *P. infestans*-specific *PiO8* element was detected by qRT-PCR. **(E)** The relative expression level of *StPRL* in VIGS downregulated potatoes was detected by qRT-PCR. **(F)** Representative images of *P. infestans* lesions on potato leaves in which StPRL was downregulated by VIGS, compared to the control. **(G)** Lesion diameter in potato leaves of StPRL downregulated by VIGS. **(H)** The relative expression level of the *P. infestans*-specific *PiO8* element was detected by qRT-PCR. The asterisk represents the position of the StPRL-GFP imprinted band. The range of disease spots on the leaves of native *N. benthamiana* is encircled. Data were collected in three biological experiments, and the Bar value represents the standard deviation. The statistical analysis was based on Student’s t test, *p < 0.05, **p < 0.01, and ***p < 0.001.

We transformed the pCa-StPRL-GFP plasmid and control pCa-GFP empty plasmid into *Agrobacterium* and transiently infected *N. benthamiana* to determine whether the resistance of *StlncRNA13558* to *P. infestans* was related to the induction of StPRL expression. Total protein was extracted from *N. benthamiana* 48* h* after infection, and StPRL was successfully expressed in tobacco leaves, as detected using western blot analysis (**Figure 4A**). *In vitro* inoculation was performed to inject *P. infestans*. The incidence of leaf infection was observed after 5 d, and the diameter of the lesions was measured. The results showed that the diameter of the lesions on the half-leaf *N. benthamiana* overexpressing StPRL was significantly smaller than that of the control (**Figures 4B, C**). qRT-PCR was performed to further clarify the abundance of *P. infestans* in the infected leaves. The results showed that the mycelial growth of StPRL-overexpressing *N. benthamiana* leaves was significantly lower than that of the control (**Figure 4D**).

We constructed a recombinant TRV2-StPRL plasmid and transferred it to *Agrobacterium*. Two-week-old potato seedlings were infected under a vacuum. Potatoes infected with the TRV2 empty plasmid were used as controls for qRT-PCR. The results showed that the expression level of StPRL was significantly lower than that in the control (**Figure 4E**). Leaves were inoculated with *P. infestans in vitro*. The disease incidence was observed, and the lesion diameter was measured after 5 d. The results showed that potato plants were susceptible to *P. infestans* after the downregulation of StPRL, and the lesion diameter was significantly larger than that of the empty control (**Figures 4F, G**). qRT-PCR results showed that the mycelial growth of *P. infestans* in potato leaves with downregulated StPRL was significantly higher than that in the control (**Figure 4H**). These results indicated that the downregulation of the potato *StPRL* gene was susceptible to *P. infestans* and that StPRL could enhance the resistance of potatoes to *P. infestans*.

### 3.5 StPRL promotes reactive oxygen species (ROS) accumulation

**Figure 5:**
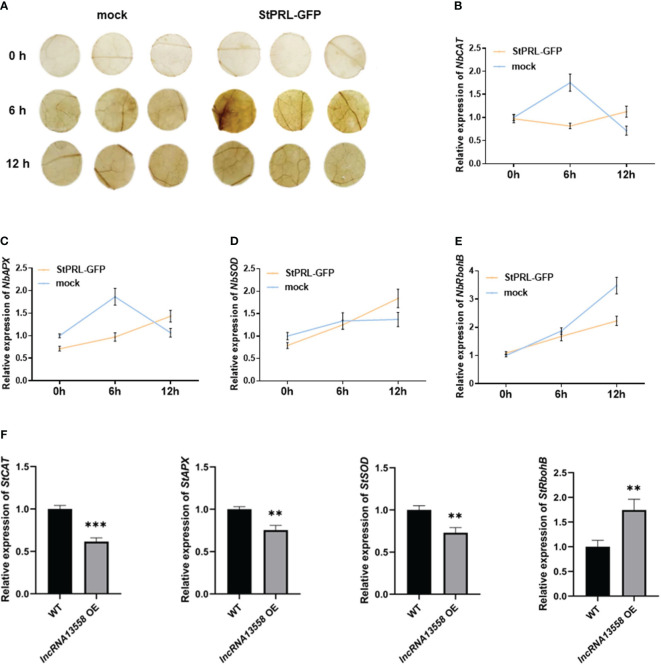
StPRL promotes ROS accumulation. **(A)** DAB staining was used to observe the accumulation of ROS in *N. benthamiana* leaves. **(B–E)** The relative expression of ROS pathway accumulation and scavenging enzyme related genes *NbCAT*, *NbAPX*, *NbSOD* and *NbRbohB* in *N. benthamiana* leaves was detected by qRT-PCR when StPRL was transiently overexpressed. **(F)** The relative expression of ROS pathway accumulation and scavenging enzyme related genes *StCAT*, *StAPX*, *StSOD* and *StRbohB* in potato leaves with stable overexpression of *StlncRNA13558* was detected by qRT-PCR. Data were collected in three biological experiments, and the Bar value represents the standard deviation. The statistical analysis was based on Student’s t test, **p < 0.01, and ***p < 0.001.

ROS are usually maintained at low levels and accumulate during plant resistance to pathogen infection (Suzuki et al., 2011; Qi et al., 2017). DAB staining is generally used to detect the accumulation of hydrogen peroxide (H_2_O_2_) in cells. pCa-StPRL-GFP was transiently overexpressed in tobacco leaves, and pCa-GFP was expressed in the other half of the tobacco leaves as the control. After 48 h, tobacco leaves were sprayed with the *P. infestans* spore suspension. ROS accumulation was observed using DAB staining 0, 6, and 12 h after spraying the plants with *P. infestans*. The results showed that the brown color of the leaves overexpressing StPRL and control leaves deepened 6 h after inoculation with *P. infestans*, with the leaves overexpressing StPRL showing a darker brown color (**Figure 5A**). The brown color in the leaves overexpressing StPRL and the control leaves was weaker after 12 h than after 6 h (**Figure 5A**). Genes related to ROS accumulation and scavenging, including *hydrogen oxide enzyme gene (NbCAT)*, *ascorbate peroxidase gene* (*NbAPX*), *superoxide dismutase gene* (*NbSOD*), and *respiratory burst oxidase gene B* (*NbRbohB*), were detected using qRT-PCR. The results showed that the expression levels of *NbCAT*, *NbAPX*, and *NbSOD* in the control group first increased and then decreased after inoculation with *P. infestans*, peaking at 6 h, whereas the expression level of *NbRbohB* gradually increased. The expression levels of *NbCAT* and *NbAPX* in the experimental group overexpressing StPRL were significantly inhibited compared to those in the control group 6 h after inoculation with *P. infestans* (**Figures 5B–E**). These results indicated that StPRL inhibited ROS scavenging.

We further detected the expression of ROS accumulation- and scavenging-related enzymes in potato plants stably overexpressing *StlncRNA13558* using qRT-PCR. The results showed that the expression levels of the ROS-scavenging enzymes *StCAT*, *StAPX*, and *StSOD* in potato plants stably overexpressing *StlncRNA13558* were inhibited. In contrast, the expression level of *StRbohB*, which promotes ROS production, increased in potato plants stably overexpressing *StlncRNA13558* (**Figure 5F**). We transiently overexpressed *StlncRNA13558* in tomato leaves, which are also a *Solanaceae* crop, to verify its broad spectrum of functions. The results showed that the expression levels of ROS synthesis- and scavenging-related genes in the transient overexpression of *StlncRNA13558* in tomato leaves were also different (**Supplementary Figure 1**). The results showed that stable overexpression of *StlncRNA13558* promoted ROS accumulation by promoting ROS production and inhibiting ROS clearance, which was consistent with the results of the transient overexpression of StPRL induced by *StlncRNA13558*.

### 3.6 StPRL activates the SA hormone pathway against *P. infestans*

**Figure 6:**
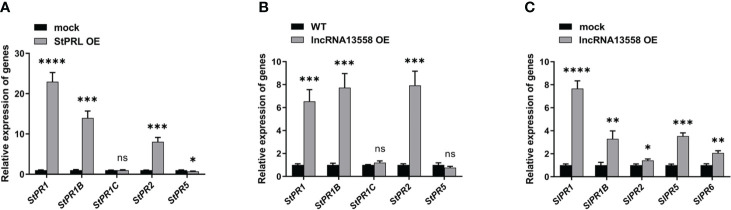
StPRL activates the SA hormone pathway against *P. infestans.* **(A)** The relative expression of *StPR1*, *StPR1B*, *StPR1C*, *StPR2*, and *StPR5* genes in the SA pathway in potato leaves with transient overexpression of StPRL was detected by qRT-PCR. **(B)** The relative expression of *StPR1*, *StPR1B*, *StPR1C*, *StPR2*, and *StPR5* genes in the SA pathway in potato leaves with stable overexpression of *StlncRNA13558* was detected by qRT-PCR. **(C)** The relative expression of *SlPR1*, *SlPR1B*, *SlPR2*, *SlPR5*, and *SlPR6* genes in the SA pathway in tomato leaves with transient overexpression of *StlncRNA13558* was detected by qRT-PCR. Data were collected in three biological experiments, and the Bar value represents the standard deviation. The statistical analysis was based on Student’s t test, ^ns^ *p* > 0.05, *p < 0.05, **p < 0.01, ***p < 0.001, and ****p < 0.0001.

PR proteins are important contributors to SAR and plant disease resistance (Hamamouch et al., 2011). The SA hormone pathway plays an important role in the plant defense response to pathogens (Waadt et al., 2022). We referred to existing transcriptome results (Cao et al., 2020) and investigated the effect of transient overexpression of StPRL on the SA hormone pathway in potatoes using qRT-PCR analysis. The results showed that StPRL overexpression significantly increased the expression of *StPR1*, *StPR1B*, and *StPR2* in the SA pathway compared to that in the control (**Figure 6A**). We further detected activation of the SA hormone pathway in potatoes stably overexpressing *StlncRNA13558* using qRT-PCR. The results showed that the expression of *StPR1*, *StPR1B* and *StPR2* in the SA pathway was significantly increased by stable overexpression of *StlncRNA13558* (**Figure 6B**). Transient overexpression of *StlncRNA13558* in tomatoes indicated that the expression of *SlPR1*, *SlPR1B*, *StPR2*, *SlPR5*, and *SlPR6* genes in the SA pathway was increased (**Figure 6C**). The results showed that the overexpression of *StlncRNA13558* activated the SA pathway, which was consistent with the transient overexpression of StPRL induced by *StlncRNA1*3558. StPRL activates the SA hormone pathway to transmit and expand resistance signals, thereby improving the overall disease resistance of plants.

## 4 Discussion

As biological regulators, lncRNAs play important roles in plant biotic stress responses by directly or indirectly regulating the expression of various mRNAs. When pathogens invade plants, the lncRNA-dependent immune system is activated to resist infection (Zaynab et al., 2018). We identified that the upregulated lncRNA *StlncRNA13558* enhanced the resistance of potatoes to *P. infestans* infection. The spatial locations of the lncRNAs relative to the protein-coding genes were diverse (Chekanova, 2021). Further studies on how *StlncRNA13558* regulates the expression of the target gene StPRL at the molecular level should be conducted.

PR proteins are important in the plant’s response to pathogen infection. We found that overexpression of *StlncRNA13558* induced the expression of StPRL, which reduced the biomass of *P. infestans* in potatoes and *N. benthamiana*. The downregulation of *StlncRNA13558* consistently led to decreased expression of StPRL, which promoted the infection of *P. infestans* in potatoes. StPRL overexpression can promote ROS accumulation in plant leaves. Some key genes in the SA pathway were upregulated in potato leaves that transiently overexpress StPRL, which, in turn, transmits and expands plant resistance to *P. infestans*. These results suggested that *StlncRNA13558* positively regulates potato resistance to *P. infestans* infection by increasing the level of its co-expressed gene, StPRL, and explained the biological mechanism by which StPRL can increase resistance to *P. infestans* infection.

Plants produce ROS to limit pathogen invasion, and H_2_O_2_ plays an important role in plant resistance to pathogen infection (Sies and Jones, 2020; Mittler et al., 2022). Rice miR528 negatively regulates rice stripe virus resistance by cutting l-ascorbate oxidase (AO) mRNA to reduce AO-mediated accumulation of ROS (Wu et al., 2017). The heavy metal transporter OsNRAMP1 in rice is induced by pathogen infection and plays an important role in plant immunity by regulating the homeostasis of metal ions and ROS (Chu et al., 2022). WSL214 is important in promoting cellular ROS homeostasis by enhancing the catalase activity and reducing photosynthetic ROS production (Wang et al., 2023). We found that *N. benthamiana* leaves that overexpressed StPRL showed deepened DAB staining spots after spraying with the *P. infestans* spore suspension. Furthermore, the expression levels of ROS-scavenging genes decreased, indicating that StPRL resisted pathogen infection by inhibiting ROS scavenging.

Following a long struggle between plants and pathogens, plants have evolved innate immune systems, including pathogen-associated molecular pattern-triggered immunity (PTI) and effector-triggered immunity (ETI) (Sun et al., 2011; Bigeard et al., 2015; Ngou et al., 2022). The PTI is a broad-spectrum basic defense response. After the start of the reaction, it usually manifests as the accumulation of reactive oxygen species, an increase in calcium ions, and the activation of plant resistance hormone pathways to resist pathogen infection successfully (Bigeard et al., 2015; Tang et al., 2017; Zhou and Zhang, 2020). After the pathogen infects the plant and activates the PTI response, it secretes effector proteins into the host cells to inhibit the PTI immune response, activating the plant ETI response (Xiang et al., 2008; Cheng et al., 2011). The plant ETI response can independently trigger the immune signaling pathway and amplify the ongoing PTI response signal (Stuart et al., 2013). The complementarity between signaling pathways in the ETI reaction was more apparent, and the intensity and persistence of the ETI reaction were higher than those of the PTI reaction (Tsuda and Katagiri, 2010). StPRL, a PR-family protein, could promote ROS accumulation and expand resistance signals by activating the SA hormone pathway. Therefore, it is speculated that StPRL may be involved in plant innate immunity; however, its specific role needs further exploration.

**Figure 7:**
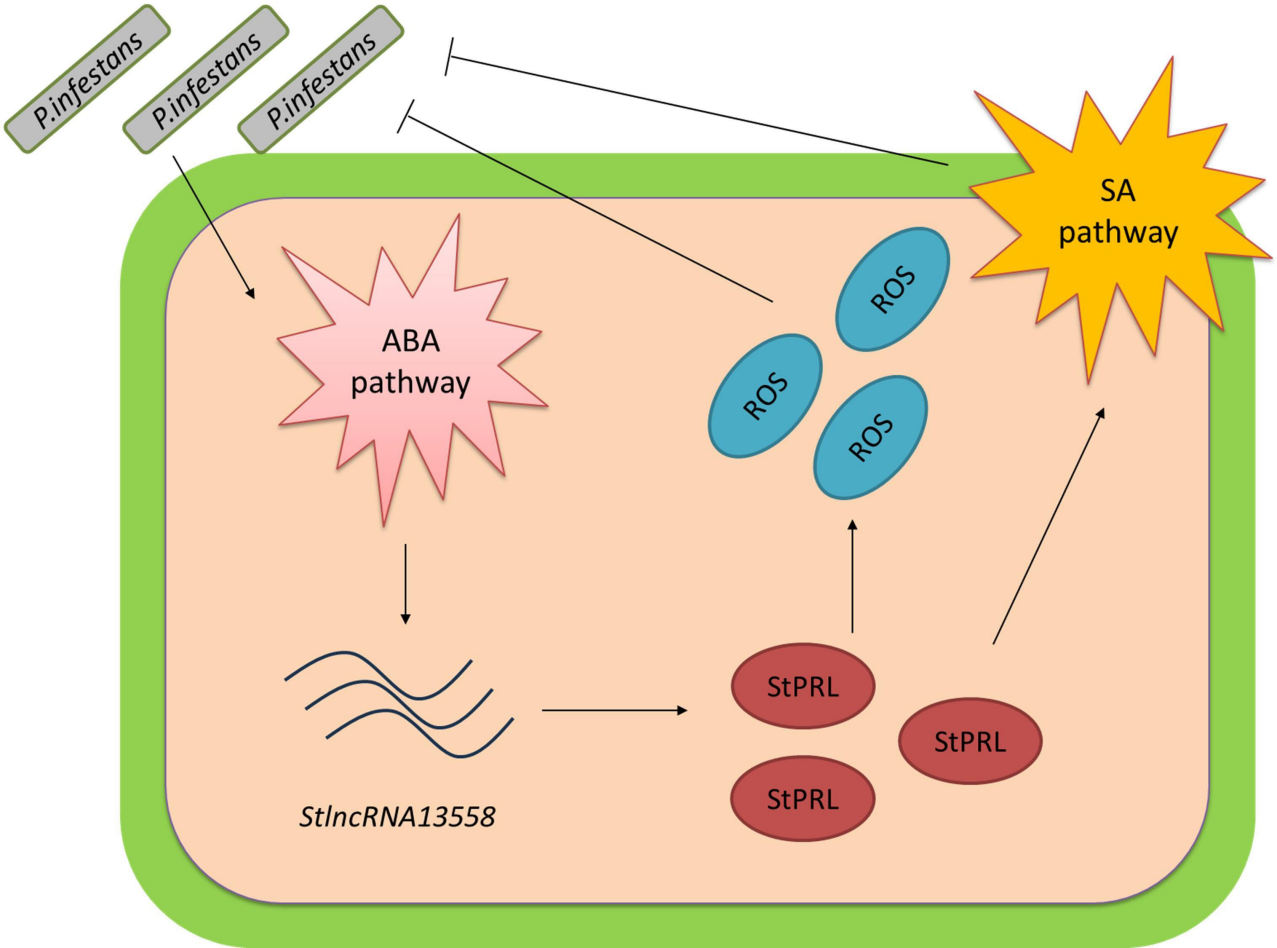
Pattern diagram of *StlncRNA13558* as the center to respond to *P. infestans* infection and improve potato resistance to *P. infestans. P. infestans* infection activates the ABA hormone pathway. The ABA pathway promotes the upregulation of *StlncRNA13558*, and *StlncRNA13558* positively regulates the expression of StPRL. The overexpression of StPRL promotes the accumulation of ROS and expands the resistance signal by activating SA hormone pathways, thereby enhancing the resistance of potatoes to *P. infestans.*.

*P. infestans* is the pathogen responsible for potato late blight, which caused a great famine in Ireland in the 18th century. We demonstrated that *P. infestans* infection activates the ABA pathway. ABA promoted the upregulation of *StlncRNA13558*, and *StlncRNA13558* positively regulated StPRL expression. Overexpression of StPRL promoted the accumulation of ROS and expanded the resistance signal via the SA pathway, thereby enhancing the resistance of potatoes to *P. infestans* (**Figure 7**). These results enrich the understanding of the immune mechanisms of plants in response to pathogen infection and provide a basis for the study of potato resistance to late blight.

## Data availability statement

Publicly available datasets were analyzed in this study. This data can be found here: The Sequence Read Archive of the National Center for Biotechnology Information (NCBI) collection was used to obtain RNA data sets of potato (PRJNA203403), which were used to identify transcripts of the potato in response to P. infestans infection.

## Author contributions

CZ: Conceptualization, Data curation, Funding acquisition, Project administration, Writing – original draft, Writing – review & editing. XZ: Conceptualization, Project administration, Writing – review & editing. KS: Formal analysis, Methodology, Project administration, Visualization, Writing – original draft, Writing – review & editing. RW: Formal analysis, Methodology, Visualization, Writing – review & editing. WC: Methodology, Visualization, Writing – review & editing. XW: Formal analysis, Writing – review & editing. YW: Formal analysis, Writing – review & editing. ZS: Formal analysis, Writing – review & editing. HL: Project administration, Writing – review & editing. SZ: Project administration, Writing – review & editing.

## Supplementary material

Supplementary Figure 1 Transient overexpression of StlncRNA13558 in tomato affects the reactive oxygen species pathway. The relative expression of ROS pathway accumulation and scavenging enzyme related genes *SlCAT*, *SlAPX*, *SlSOD* and *SlRboh* in tomato leaves with transient overexpression of *StlncRNA13558* was detected by qRT-PCR. Data were collected in three biological experiments, and the Bar value represents the standard deviation. The statistical analysis was based on Student’s t test, ^ns^ *p* > 0.05 and ***p* < 0.01.
