# Supplementary figures and images for: Abscisic-acid-responsive StlncRNA13558 induces StPRL expression to increase potato resistance to Phytophthora infestans infection
Source: Front Plant Sci. 2024 Mar 5;15:1338062. doi: 10.3389/fpls.2024.1338062 (PMC10948444; doi:10.3389/fpls.2024.1338062)

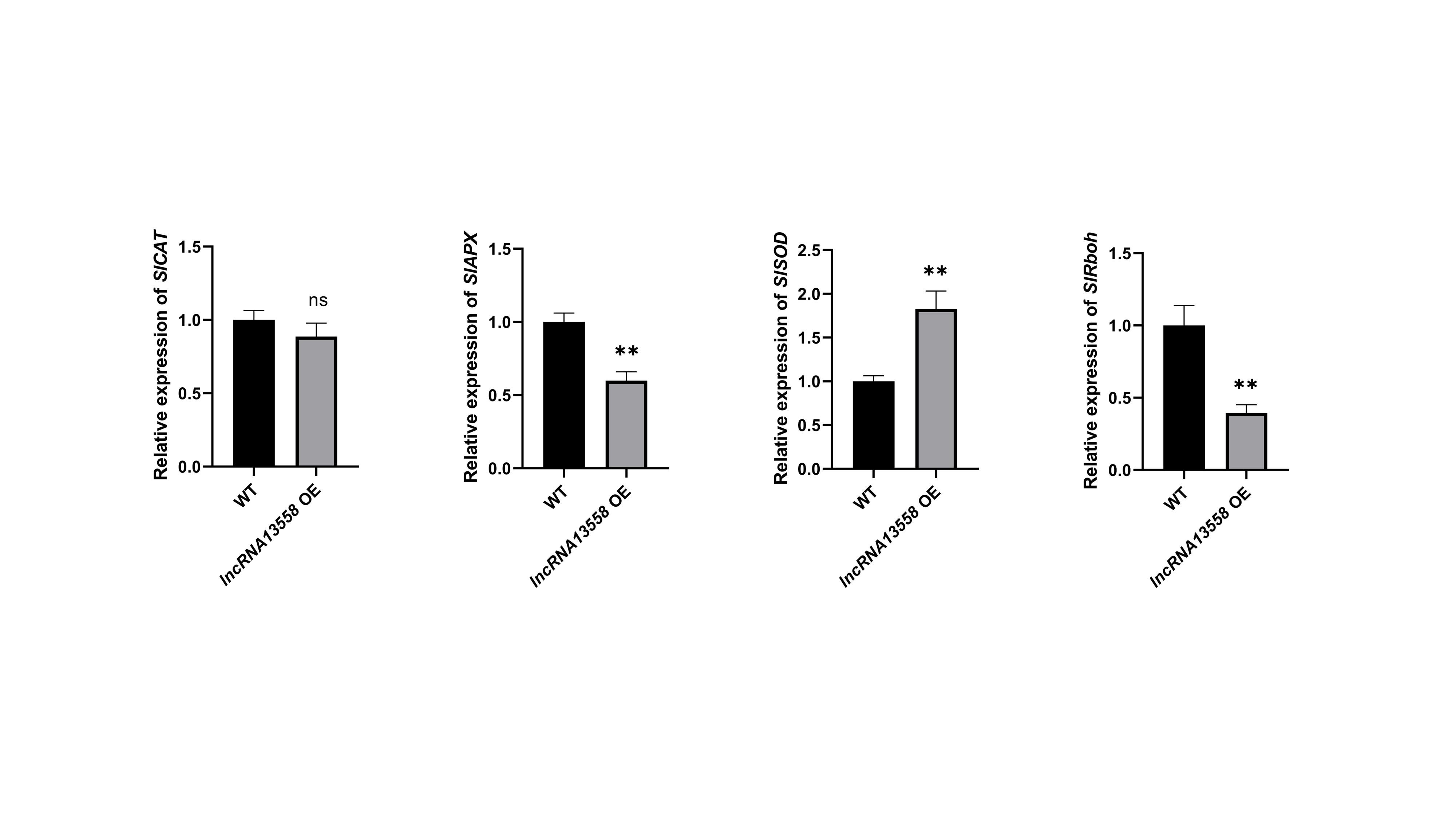

Supplement: Supplementary Figure 1 — Transient overexpression of StlncRNA13558 in tomato affects the reactive oxygen species pathway. The relative expression of ROS pathway accumulation and scavenging enzyme related genes SlCAT, SlAPX, SlSOD and SlRboh in tomato leaves with transient overexpression of StlncRNA13558 was detected by qRT-PCR. Data were collected in three biological experiments, and the Bar value represents the standard deviation. The statistical analysis was based on Student’s t test, ns p > 0.05 and **p < 0.01. [file Image_1.jpeg]
